# Anti-inflammatory effect of bergamot leaves extract attenuates cardiac remodeling in obese rats by regulating the protein expression of the collagen/metalloproteinase axis

**DOI:** 10.1371/journal.pone.0334015

**Published:** 2025-10-24

**Authors:** Taynara Aparecida Vieira, Juliana Silva Siqueira, Erika Tiemi Nakandakare-Maia, Nubia Alves Grandini, Marina Gaiato Monte, Giovanna Baron, Dijon Henrique Salomé de Campos, Giancarlo Aldini, Silmeia Garcia Zanati Bazan, Fabiane Valentini Francisqueti-Ferron, Lilian Cristina Pereira, Marina Politi Okoshi, Francis Lopes Pacagnelli, Artur Junio Togneri Ferron, Camila Renata Corrêa

**Affiliations:** 1 São Paulo State University (UNESP), Medical School, Botucatu, São Paulo, Brazil; 2 Department of Pharmaceutical Sciences, University of Milan, Milan, Italy; 3 Integrated Colleges of Bauru (FIB), Bauru, São Paulo, Brazil; 4 São Paulo State University (UNESP), School of Agricultural Sciences, Botucatu, São Paulo, Brazil; 5 Department of Physiotherapy, University of Western São Paulo (UNOESTE), Presidente Prudente, São Paulo, Brazil; University of Michigan, UNITED STATES OF AMERICA

## Abstract

Inflammation is associated with the pathogenesis of obesity-related disorders, including cardiac remodeling. Bergamot is known for its promising anti-inflammatory effects. Thus, this study aimed to investigate the anti-inflammatory effect of bergamot leaves extract (BLE) in attenuating cardiac remodeling in obese rats through the regulation of protein expression of the collagen/metalloproteinase axis. Male Wistar rats were fed a control diet (C) or a high sugar-fat diet (HSF) for 20 weeks. After developing cardiac remodeling, the animals were again distributed into three groups to receive BLE (50 mg/kg/day) or placebo (water) via gavage for 10 weeks: C, HSF and HSF + BLE. The HSF group exhibited obesity (HSF 8.77 ± 2.64 *vs* C 3.09 ± 1.02, *p = *0.007), dyslipidemia (HSF 94.4 ± 19.1 *vs* C 26.7 ± 5.2, *p < *0.001), hypertension (HSF 141 ± 8 *vs* C 120 ± 4, *p = *0.001), insulin resistance (HSF 6.91 ± 1.38 *vs* C 2.47 ± 1.01, *p < *0.001), cardiac remodeling and dysfunction, cardiac inflammation, decreased metalloproteinase-2 (MMP-2) (HSF 0.43 ± 0.09 *vs* C 0.71 ± 0.07, *p = *0.009) and increased type III collagen (HSF 1.32 ± 0.27 *vs* C 1.00 ± 0.18, *p = *0.038). In contrast, BLE was effective in improving dyslipidemia (HSF + BLE 55.2 ± 9.7 *vs* HSF 94.4 ± 19.1, *p < *0.001), insulin resistance (HSF + BLE 3.79 ± 0.76 *vs* HSF 6.91 ± 1.38, *p < *0.001), cardiac remodeling and function, as well as inflammation, MMP-2 (HSF + BLE 0.84 ± 0.22 *vs* HSF 0.43 ± 0.09, *p < *0.001) and type III collagen (HSF + BLE 0.68 ± 0.11 *vs* HSF 1.32 ± 0.27, *p < *0.001) in the HSF group. Therefore, the anti-inflammatory effect of BLE improved cardiac remodeling in obese rats through the regulation of protein expression of the collagen/metalloproteinase axis.

## Introduction

Excess body fat causes cardiac abnormalities such as hemodynamic, morphological and functional changes, by different mechanisms, associated with the release of pro-inflammatory cytokines and collagen protein expression [1–4]. In situations of excessive nutrient intake, there is hypertrophy of the adipose tissue that initially triggers a local inflammatory response, characterized by increased synthesis of pro-inflammatory cytokines, such as tumor necrosis fator-alpha (TNF-α) and interleukin-6 (IL-6), which are strongly involved in the etiology of obesity-related cardiomyopathy. Recent studies from our research group demonstrate that this condition culminates in the cardiac production of inflammatory cytokines through the activation of the Toll-like pathway 4 (Toll-like receptor 4, TLR-4) [2,3,5].

Collagen is a component of the cardiac extracellular matrix (ECM), mainly types I and III, important for the maintenance of cardiac architecture and function. In contexts with alterations in the myocardial interstitial collagen, as a result of increased synthesis or a decreased degradation, there occurs an increase in tissue stiffness, culminating in cardiac remodeling, well established in pressure and volume overload models [4,6,7].

Alteration and/or impairment in cardiac performance occur with the increase in pro-inflammatory cytokines and interstitial collagen, the mechanisms related to the modulation of these responses are not well established in obesity models. One of the possibilities is the involvement of matrix metalloproteinases (MMPs), proteolytic enzymes responsible for degradation of collagen fibers. MMPs can be modulated by pro-inflammatory cytokines in different models [7–13] in particular MMP-2, the most abundant in the myocardium and related to the remodeling process [14].

Thus, considering the importance of inflammation in the pathogenesis of obesity-related disorders, including cardiac remodeling and dysfunction, the search for anti-inflammatory therapeutic strategies presents promising possibilities. Bergamot (*Citrus bergamia*), is a citrus fruit used for pharmaceutical purposes in the production of essential oil, which differs from the others not only by the composition of its profile of flavonoids present in the fruit juice, but also by the particularly high content of these compounds, which has aroused scientific interest because they perform hypolipemic, hypoglycemic, anti-inflammatory and antioxidant activities, proving to be effective in the treatment of the parameters that make up the metabolic syndrome [15–17]. Recently, some investigations have shown a higher concentration of phenolic compounds in the leaves compared to the fruit, indicating a greater therapeutic potential [15,18].

Therefore, in view of the scarcity of studies that address the anti-inflammatory effect of bergamot leaves extract (BLE) in the treatment of heart diseases associated with obesity, the aim of the present study was to investigate the anti-inflammatory effect of BLE in attenuating cardiac remodeling in obese rats through the regulation of protein expression of the collagen/metalloproteinase axis.

## Materials and methods

### Experimental protocol

All procedures involving animals were in compliance with the National Institute of Health’s Guide for the Care and Use of Laboratory Animals [19], and ethical approval was granted by the Ethics Committee of São Paulo State University (1393/2021) approved on 28 September 2021.

### Groups characterization

The sample size of the present study was calculated based on the work on myocardial collagens I and III of obese rats fed a saturated high-fat diet [20]. Thus, a sample of 24 animals was estimated with a sampling power of 90%, a two-tailed alpha of 0.05, and a sample loss of approximately 25%. Male Wistar rats (±187 g, n = 24) were kept in an environmentally controlled room (22 °C ± 3 °C; 12 h light-dark cycle and relative humidity of 60 ± 5%) and randomly distributed into 2 experimental groups by 20 weeks. During this period, the animals received ad libitum water and a control diet (C, n = 8) or a high sugar-fat diet (HSF, n = 16) containing 25% sucrose. The diets were prepared in our laboratory as previously published [21]. After this period, echocardiography was performed on the animals, and a 95% confidence interval for cardiac remodeling was constructed based on morphological, systolic, and diastolic variables in the HSF and C groups. This criterion is important because animals subjected to different dietary models do not always exhibit the expected response. This can lead to misclassification of animals and, consequently, false conclusions [22]. Therefore, the HSF animals that did not develop cardiac remodeling were excluded from the experiment, and two animals in the control group died during the experiment due to gavage. After, the animals were redivided into 3 groups to begin treatment with BLE by gavage daily (50 mg/kg) for 10 weeks: control diet + placebo (C, n = 6), HSF diet + placebo (HSF, n = 6), HSF + BLE (n = 6). The nutritional composition of both diets is presented in Table 1.

**Table 1:** Diet composition and nutritional values.

Components	Control	HSF
Soybean meal (g/kg)	335	340
Sorghum (g/kg)	278	80
Soy hulls (g/kg)	188	116
Dextrin (g/kg)	146	20
Sucrose (g/kg)	–	80
Fructose (g/kg)	–	180
Soybean oil (g/kg)	14	–
Lard (g/kg)	–	154
Minerals (g/kg)	25	25
Salt (g/kg)	4	8
**Nutritional values**	–	–
Protein (% of ingredients)	20.0	18.0
Carbohydrate (% of ingredients)	60.0	53.5
Fat (% of ingredients)	4.00	16.5
% of unsaturated	69.0	47.0
% of saturated	31.0	53.0
% Energy from protein	22.9	16.6
% Energy from carbohydrate	66.8	49.2
% Energy from fat	10.4	34.2
Energy (kcal/g)	3.59	4.35

HSF, high sugar-fat diet.

At the end of 30 weeks, the animals were fasted for 8 h and then anesthetized with thiopental (120 mg/Kg/i.p.) and euthanized by decapitation after verification of the absence of palpebral, foot, interdigital and caudal reflexes. Blood samples were collected, and the plasma was separated by centrifugation (800 × *g* at 4 °C for 10 min) for metabolic analyses. The adipose tissue was isolated, dissected, and weighed for nutritional parameter assessment and cardiac tissue was also collected from each animal for further analysis.

### BLE

Bergamot leaves were harvested in a farm located in the Reggio Calabria region, Italy, and the dry extract was obtained at H&AD (Herbal & Antioxidant Derivatives S.r.l.) plant located in Localit`a Chiusi, 89032 Bianco (RC), Italy (www.head-sa.com). The processes involved in the extraction and administration were described and published by our research group [23]. The dosage was defined according to published fruit juice data [24].

### Nutritional and metabolic parameters

The nutritional parameters included the following parameters: chow fed, water intake, caloric intake, final body weight (FBW) and adiposity index. Caloric intake was determined by multiplying the energy value of each diet (g × Kcal) by the daily food consumption. For the HSF group, caloric intake also considered the calories from water (0.25 × 4 × mL consumed). After euthanasia, the fat deposits (visceral (VAT), epididymal (EAT) and retroperitoneal (RAT)) were used to calculate the adiposity index by the following formula [25]:


[(VAT + EAT + RAT)/FBW×100.

Triglycerides levels were measured using specific kits (BIOCLIN^®^, Belo Horizonte, MG, Brazil) and analyzed by a colorimetric-enzymatic method in an automatic enzymatic analyzer system (Chemistry Analyzer BS-200, Mindray Medical International Limited, Shenzhen, China). The homeostatic model of insulin resistance (HOMA-IR) was used as an insulin resistance index, calculated according to the formula [26]:


$$HOMA-IR=\left(fasting\;glucose\;\left(\frac{mmol}{L}\right)\times fasting\;insulin\;\left(\frac{\mu U}{mL}\right)\right)/22.5.$$

Glucose levels were measured in a blood drop using a handheld glucometer (Accu-Chek Performa, Roche Diagnostics Brazil Limited, São Paulo, Brazil), while insulin levels were assessed by an enzyme-linked immunosorbent assay (ELISA) method using commercial kits (Linco Research Inc., R&D Systems, Millipore and B-Brigde International Inc.) and the reading was performed in a Spectramax 190 microplate spectrophotometer (Molecular Devices^®^, Sunnyvale, CA, USA).

### Systolic blood pressure (SBP)

Evaluation was assessed in conscious rats by the non-invasive tail-cuff method with a NarcoBioSystems® Electro-Sphygmomanometer (International Biomedical, Austin, TX, USA). The animals were kept in a wooden box (50 × 40 cm) between 38 and 40 °C for 4–5 min to stimulate arterial vasodilation [27]. After this procedure, a cuff with a pneumatic pulse sensor was attached to the tail of each animal. The cuff was inflated to 200 mmHg pressure and subsequently deflated. The blood pressure values were recorded on a Gould RS 3200 polygraph (Gould Instrumental, Valley View, OH, USA). The average of three pressure readings was recorded for each animal.

### Echocardiographic study

Doppler echocardiographic evaluation was performed by a single examiner at the 20th and 30th weeks. Animals were anesthetized with ketamine (50 mg/kg, i.p.) and xylazine hydrochloride (1 mg/kg, i.p.). After trichotomy of the anterior chest region, the animals were placed in slight left lateral decubitus for the exam. The equipment used was model Vivid S6 (General Electric Medical Systems, Tirat Carmel, Israel) with a multifrequency ultrasonic transducer 5.0–11.5 MHz. To implement structural measurements of the heart, the images were obtained in one-dimensional mode (M-mode) guided by the images in two-dimensional mode with the transducer in the parasternal position, minor axis [23].

Left ventricular (LV) evaluation was performed by positioning the cursor M-mode just below the mitral valve plane at the level of the papillary muscles. The following cardiac structures were used to analyze cardiac morphology: left ventricular diastolic diameter (LVDD), posterior wall diastolic thickness (PWDT), interventricular septum diastolic thickness (IVSDT), left ventricular mass index (LVM index) and relative thickness of the left ventricular (LVRT). The systolic LV function was assessed by the following parameters: ejection fraction (EF) and posterior wall shortening velocity (PWSV). The LV diastolic function was evaluated using the following indices: isovolumic relaxation time (IVRT), E wave deceleration time (EWDT) and E/E′ ratio.

### Cardiac inflammatory markers

Cardiac tissue samples were homogenized and centrifuged as already described by our research group [2,3]. The levels of TNF-α and IL-6 were evaluated using the ELISA method using commercial kits from R&D System, Minneapolis, USA. The results were corrected by the protein amount.

### Myocardial MMP-2 activity

The activity was determined by the zymography technique as described by Tyagi et al. [28]. In brief, samples were diluted in extraction buffer with 50 mM of Tris pH 7.4, 0.2 M of sodium chloride (NaCl), 0.1% of Triton X, and 10 mM of calcium chloride (CaCl_2_). Protein in samples was quantified by the Bradford method [29]. Samples with 20 µg of protein were then diluted in application buffer with 0.5 M of Tris pH 6.8, 50% of glycerol, and 0.05% of bromophenol blue, and loaded into wells of 8% of sodium dodecyl sulfate (SDS) polyacrylamide containing 1% gelatin. Electrophoresis was run in a Bio-Rad apparatus at 80 V for 2 h. Gel was removed, washed with 2.5% of Triton X-100, and washed with 50 mM of Tris pH 8.4. Gel was then incubated at 37 °C overnight in activation solution with 50 mM of Tris pH 8.4, 5 mM of CaCl_2_, and zinc chloride (ZnCl_2_). Staining was performed for 2 h with 0.5% of comassie blue and destaining in 30% of methanol and 10% of acetic acid at room temperature on a rotatory shaker. To identify the gel activity of MMP-2, we used recombinant mouse/rat MMP-2 standard (R&D Systems) as a positive control. The gels were photographed by Gel Logic 6000 Pro (Carestream Health Inc.) and the intensity of gelatinolytic action (clear bands) was analyzed by GelPro 3.1 [30].

### Myocardial type III collagen protein expression

LV samples were homogenized in RIPA buﬀer with protease and phosphatase cocktail inhibitors, and centrifuged for 20 min at 4 °C, for the supernatant collection. Protein concentration was determined by the Bradford method [29] and samples were diluted in Laemmli buﬀer and loaded (50 μg of protein) into a 10% SDS–polyacrylamide gel. Electrophoresis was performed in a Mini-Protean 3 Eletrophoresis Cell system (Bio-Rad, Hercules, USA), at 4 °C, at 50 V for 30 min and then at 120 V for 2 h. After that, the gels were transferred to a nitrocellulose membrane using Trans-Blot Turbo-Transfer System (BioRad) in a semi-wet transfer for 30 min at 25 V and 1.0 A, using transfer buffer (25 mM Tris, 192 mM glycine, 20% methanol and 0.1% SDS). Next, the membranes were blocked in 5% bovine serum albumin solution at room temperature for 2 h in order to prevent unspecific bindings with antibodies and washed with basal solution (Tris 1 M, NaCl 2.5 M and Tween 20), pH 8.0. Incubation with primary antibodies was performed overnight at 4 °C in Tris-buﬀered saline solution containing Tween 20 (TBS-T) and 3% bovine serum albumin [5]. Antibody dilutions were 1:1000 for anti-collagen III (rabbit monoclonal antibody, ABCAM ab184993) and 1:1000 for anti-beta actin (rabbit polyclonal antibody, ABCAM ab8227). After incubating overnight at 4 °C in TBS-T containing 1% bovine serum albumin with the Abcam secondary antibodies (dilutions 1:5000 for goat anti-rabbit IgG H&L (HRP) (ABCAM ab205718)), proteins were revealed using the chemiluminescence method according to the manufacturer’s instructions (ECL SuperSignal^®^ West Pico Chemiluminescent Substrate, Thermo Scientiﬁc). Band intensities were evaluated by ImageQuant TL 1D Version 8.1 (GE Healthcare Life Sciences). β-actin protein was used as an endogenous control.

### Statistical analysis

Data are presented as means ± standard deviation or medians (interquartile range). Differences among the groups were determined by one-way ANOVA followed by *post hoc* Tukey’s test for parametric data or Kruskal-Wallis followed by *post hoc* Tukey’s test for non-parametric data. Statistical analyses were performed using Sigma Stat for Windows Version 3.5. (Systat Software, Inc., San Jose, CA, USA). A *p* value < 0.05 was considered as statistically significant.

## Results

The nutritional and metabolic parameters are shown in Table 2. The HSF group showed increased water intake, caloric intake, final body weight, adiposity index, triglycerides and insulin resistance; however, chow intake was reduced when compared to the C group. The HSF + BLE group had improvement in insulin resistance, as indicated by HOMA-IR, and triglyceride levels compared to the HSF group.

**Table 2:** Nutritional and metabolic analysis.

Variables	C	HSF	HSF + BLE
**Chow fed (g/day)**	23.2 ± 2.4	13.1 ± 1.7*	12.6 ± 1.2*
**Water intake (mL/day)**	34.2 ± 2.7	51.6 ± 3.6*	49.5 ± 5.8*
**Caloric intake (kcal/day)**	84.5 ± 8.6	108.6 ± 6.0*	104.1 ± 10.2*
**Final body weight (g)**	448 ± 39	618 ± 72*	575 ± 62*
**Adiposity index (%)**	3.09 ± 1.02	8.77 ± 2.64*	7.92 ± 3.75*
**Triglycerides (mg/dL)**	26.7 ± 5.2	94.4 ± 19.1*	55.2 ± 9.7^#^*
**HOMA-IR**	2.47 ± 1.01	6.91 ± 1.38*	3.79 ± 0.76^#^

Data expressed as means ± standard deviation (6 animals/group). Comparison by one-way ANOVA with Tukey’s *post-hoc*. C, Control diet+placebo; HSF, high sugar-fat diet+placebo; HSF + BLE, high sugar-fat diet+ bergamot leaves extract. *p <* 0.05.

* *vs* C.

# *vs* HSF.

Table 3 shows the cardiovascular parameters. The HSF group presented cardiac remodeling characterized by changes in structural variables (LVDD, PWDT, IVSDT, LVM index, LVRT), cardiac systolic and diastolic function (EF, PWSV, IVRT, EWDT and E/E’ ratio), and increased SBP. Treatment with BLE attenuated cardiac remodeling and recovered systolic and diastolic functions compared to the HSF group.

**Table 3:** Cardiovascular parameters.

Variables	C	HSF	HSF + BLE
**LVDD (mm)**	6.90 ± 0.28	7.79 ± 0.34*	7.43 ± 0.03*
**PWDT (mm)**	1.49 ± 0.05	1.90 ± 0.11*	1.53 ± 0.02^#^
**IVSDT (mm)**	1.54 ± 0.03	2.02 ± 0.15*	1.58 ± 0.05^#^
**LVM index**	1.47 ± 0.23	2.03 ± 0.36*	1.38 ± 0.15^#^
**LVRT**	0.43 ± 0.02	0.49 ± 0.04*	0.41 ± 0.01^#^
**EF (%)**	0.94 ± 0.02	0.90 ± 0.02*	0.94 ± 0.01^#^
**PWSV (mm/s)**	83.8 ± 6.3	60.8 ± 11.2*	76.3 ± 4.7^#^
**IVRT (ms)** ^**a**^	22.0 (23.0-22.0)	31.5 (37.0-28.2)*	21.0 (23.2-20.0)^#^
**EWDT (ms)**	43.5 ± 0.8	55.5 ± 8.1*	44.8 ± 3.9^#^
**E/E’ ratio**	13.7 ± 1.3	22.6 ± 4.7*	14.1 ± 1.2^#^
**SBP (mmHg)**	120 ± 4	141 ± 8*	139 ± 11*

LV, left ventricular; LVDD, left ventricular diastolic diameter; PWDT, posterior wall diastolic thickness; IVSDT, interventricular septum diastolic thickness; LVM index, left ventricular mass index; LVRT, relative thickness of the left ventricular; EF, ejection fraction; PWSV, posterior wall shortening velocity; IVRT, isovolumic relaxation time; EWDT, E wave deceleration time; SBP, systolic blood pressure. Data expressed as means ± standard deviation and ^a^ median (interquartile range) (6 animals/group). Comparison by one-way ANOVA or ^a^ Kruskal-Wallis with Tukey’s *post hoc*. C, Control diet+placebo; HSF, high sugar-fat diet+placebo; HSF + BLE, high sugar-fat diet+ bergamot leaves extract. **p <* *0.05.

* *vs* C.

# *vs* HSF.

**Fig 1:**
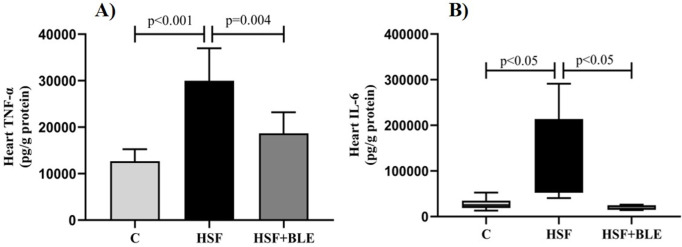
Cardiac inflammatory parameters. (A) tumoral necrosis factor – alpha (TNF-α); (B) Interleukin- 6 (IL-6). Data are expressed in mean ± standard deviation or median (min-max) (6 animals/group). Comparison by one-way ANOVA or Kruskal-Wallis with Tukey’s *post hoc*. *p < *0.05. C, Control diet+placebo; HSF, high sugar-fat diet+placebo; HSF + BLE, high sugar-fat diet+ bergamot leaves extract.

Fig 1 shows cardiac inflammatory parameters. TNF-α (Fig 1A) and IL-6 (Fig 1B) levels were higher in HSF than in the C group, representing an inflammatory state. The treatment with BLE attenuated the concentration of cardiac inflammatory parameters in the HSF + BLE group compared to the HSF group.

**Fig 2:**
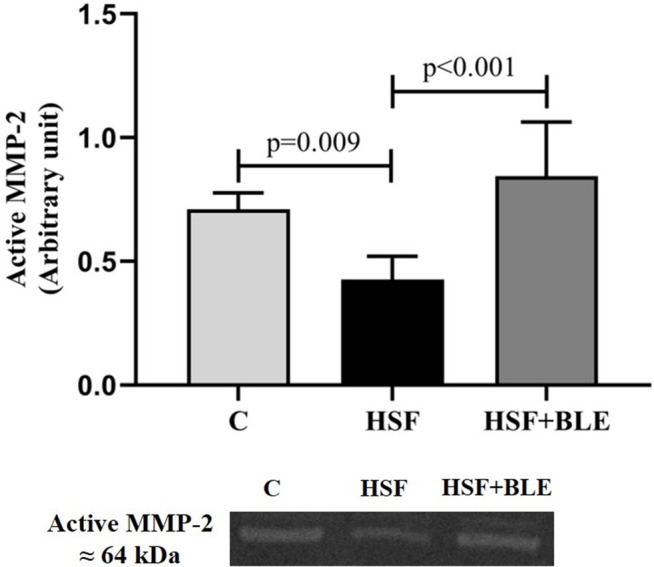
MMP-2 activity. Data are expressed in mean ± standard deviation (6 animals/group). Comparison by one-way ANOVA with Tukey’s *post-hoc*. *p < *0.05. C, Control diet+placebo; HSF, high sugar-fat diet+placebo; HSF + BLE, high sugar-fat diet+ bergamot leaves extract.

Fig 2 (S1 Fig Raw image) shows MMP-2 activity. The HSF had lower MMP-2 activity than the C group. Supplementation with BLE increased the MMP-2 activity compared to the HSF group.

**Fig 3:**
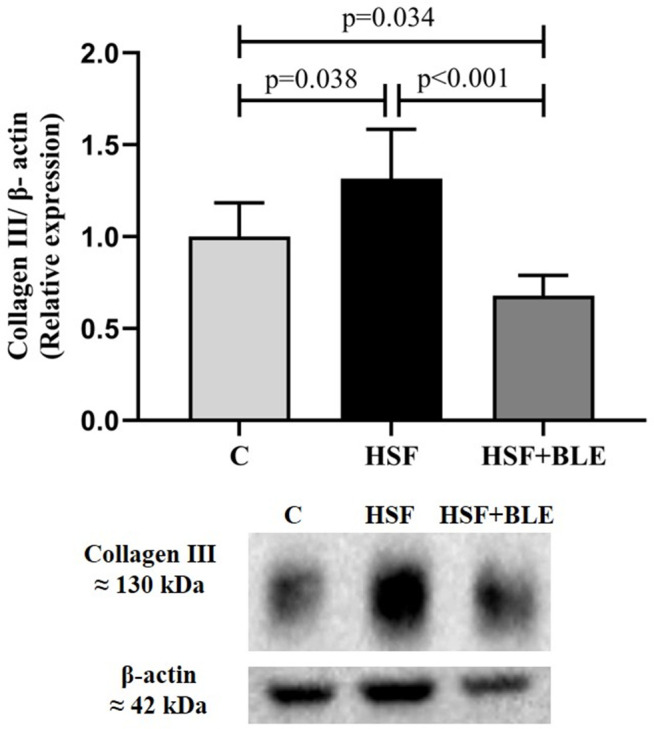
Relative expression of collagen III/β-actin. Data are expressed in mean ± standard deviation (6 animals/group). Comparison by one-way ANOVA with Tukey’s *post-hoc*. *p <* 0.05. C, Control diet+placebo; HSF, high sugar-fat diet+placebo; HSF + BLE, high sugar-fat diet+ bergamot leaves extract.

Fig 3 (S2 Fig Raw image) shows the myocardial expression of collagen III/β-actin. It can be observed that the HSF group presented higher protein expression compared to the C group. On the other hand, BLE supplementation reduced collagen expression in the HSF + BLE group compared to the HSF and C groups.

## Discussion

According to the literature, diets rich in fats and carbohydrates may be responsible for the pandemic of obesity and disorders related to excess body fat, such as insulin resistance, type 2 diabetes mellitus, high blood pressure and dyslipidemia [31,32]. The findings of the present study corroborate the literature, since the HSF group developed obesity, dyslipidemia, systolic arterial hypertension and insulin resistance (Tables 2 and 3), showing the effectiveness of the dietary model in providing obesity and its related disorders [21]. In contrast, HSF animals supplemented with BLE had improved insulin resistance and triglyceride levels. This improvement can be attributed to the high concentration of polyphenols present in bergamot, since both bergamot fruit and leaves extract share the same class of polyphenolic compounds, some of which are in higher concentrations in the leaves, and show promising results due to their antioxidant, anti-inflammatory, and hypolipidemic effects that culminate in improved glucose uptake and lipid metabolism [15,23,33].

Several studies, both in experimental models and humans, have sought to establish a dietary model capable of inducing cardiac remodeling and dysfunction [34]. This condition was confirmed in this study, since the HSF group showed cardiac remodeling (through an increase in LVDD, PWDT, IVSDT, LVM index and LVRT), accompanied by impairment of systolic and diastolic ventricular function (through a decrease in EF, PWSV and increase in IVRT, EWDT and E/E’ ratio) (Table 3). However, the mechanisms underlying cardiac remodeling are not yet fully understood, therefore, this study aimed to investigate the anti-inflammatory effect of BLE in attenuating cardiac remodeling in obese rats through the regulation of protein expression of the collagen/metalloproteinase axis. The results showed that BLE supplementation attenuated cardiac remodeling and recovered systolic and diastolic function (Table 3). Remodeling induced by obesity can be explained by several mechanisms [5,7]. These include chronic and low-grade inflammatory states characterized by increased production of inflammatory cytokines, such as TNF-α and IL-6 [2,3,5,35]. Cytokines are involved in important cardiac processes such as myocyte hypertrophy, ventricular remodeling and left ventricular dysfunction [36]. Our findings confirm the evidence of the association between cardiac remodeling and inflammation, since the cardiac levels of TNF-α and IL-6 were higher in the HSF group (Fig 1A and 1B). The HSF + BLE group had recovery of cardiac alterations concomitant with a lower level of inflammatory cytokines, confirming the anti-inflammatory potential of BLE.

One of the hypotheses explaining the involvement of inflammatory cytokines in the cardiac remodeling process is their ability to modulate collagen deposition via the activity of matrix MMPs. MMPs play a key role in cardiac collagenolytic activity, especially the MMP-2, which degrades interstitial collagen types I and III, responsible for cardiac rigidity [6,37]. In our study, the HSF group had a reduction in MMP-2 activity (Fig 2) and an increase in the protein expression of collagen III (Fig 3). Supplementation with leaves extract increased MMP-2 activity and decreased collagen expression, confirming the ability of inflammatory cytokines to modulate MMP-2 activity and its subsequent regulation of interstitial collagen.

Different experimental models have been used to investigate the participation of the MMP-collagen pathway in cardiac remodeling [8,9,38–40]. However, few studies have investigated the participation of this pathway in obesity models [8,41]. The studies that analyzed protein expression of collagen I and III in the heart of obese animals [8,41,42] showed alterations in collagen I with no changes in the expression of collagen III [42] or increase in both types of collagen [8,41].

The scarcity and divergences between the works presented in the literature strengthen the importance of this work. The findings confirm our hypothesis that BLE exerts a direct action on the heart, reducing cardiac levels of TNF-α and IL-6. This anti-inflammatory effect is reflected in increased MMP-2 activity, which culminates in reduced protein expression of type III collagen and results in improved cardiac remodeling. Future studies are needed to address the mechanisms by which inflammatory cytokines modulate the activity of metalloproteinases in cardiac remodeling during obesity. Additionally, it is relevant to include female models in future studies to evaluate whether BLE has comparable or distinct effects.

## Conclusion

The anti-inflammatory effect of BLE improved cardiac remodeling in obese rats through the regulation of protein expression of the collagen/metalloproteinase axis.

## Supporting information

S1 Fig **Raw image 1.** Original gels for Fig 2.
(PDF)

S2 Fig **Raw image 2.** Original blots for Fig 3.
(PDF)

S1 Data Minimal data set. The minimal data set underlying the findings reported in our study.
(XLSX)
