# Supplementary figures and images for: Anti-inflammatory effect of bergamot leaves extract attenuates cardiac remodeling in obese rats by regulating the protein expression of the collagen/metalloproteinase axis
Source: PLoS One. 2025 Oct 24;20(10):e0334015. doi: 10.1371/journal.pone.0334015 (PMC12551895; doi:10.1371/journal.pone.0334015)

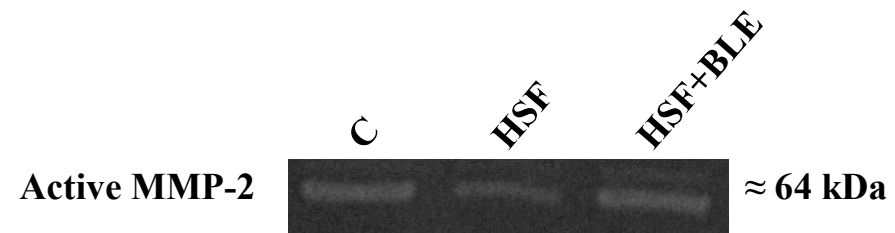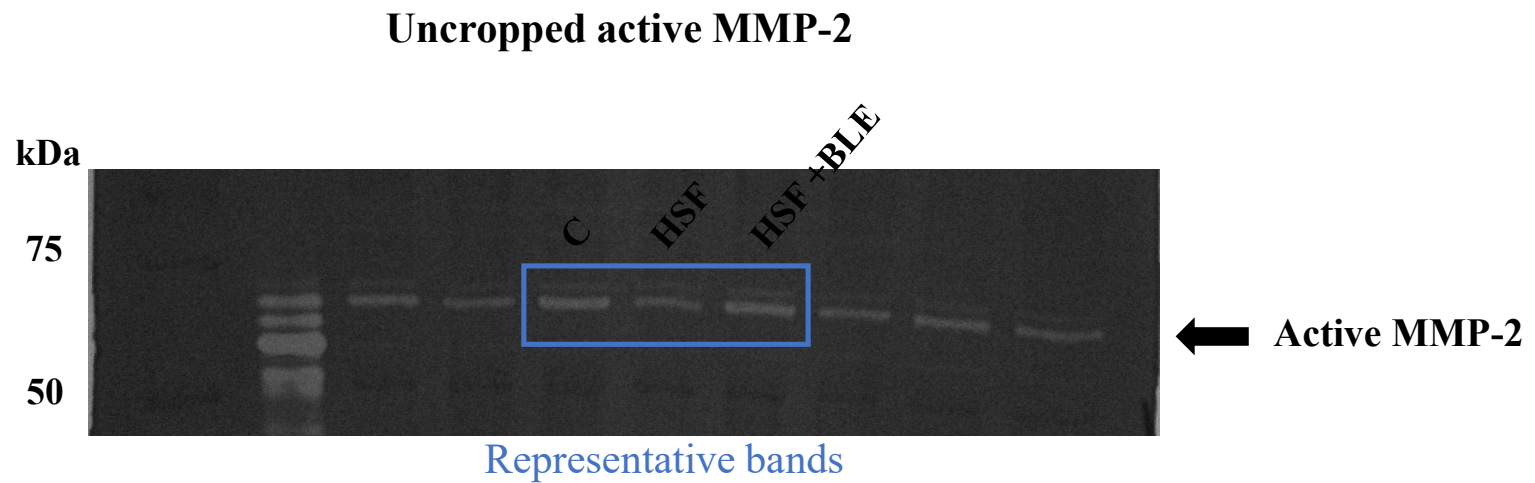

S1 Fig. Raw image 1. Original gels for Fig 2

Supplement: S1 Fig — Raw image 1. Original gels for Fig 2. (PDF) [file pone.0334015.s001.pdf]

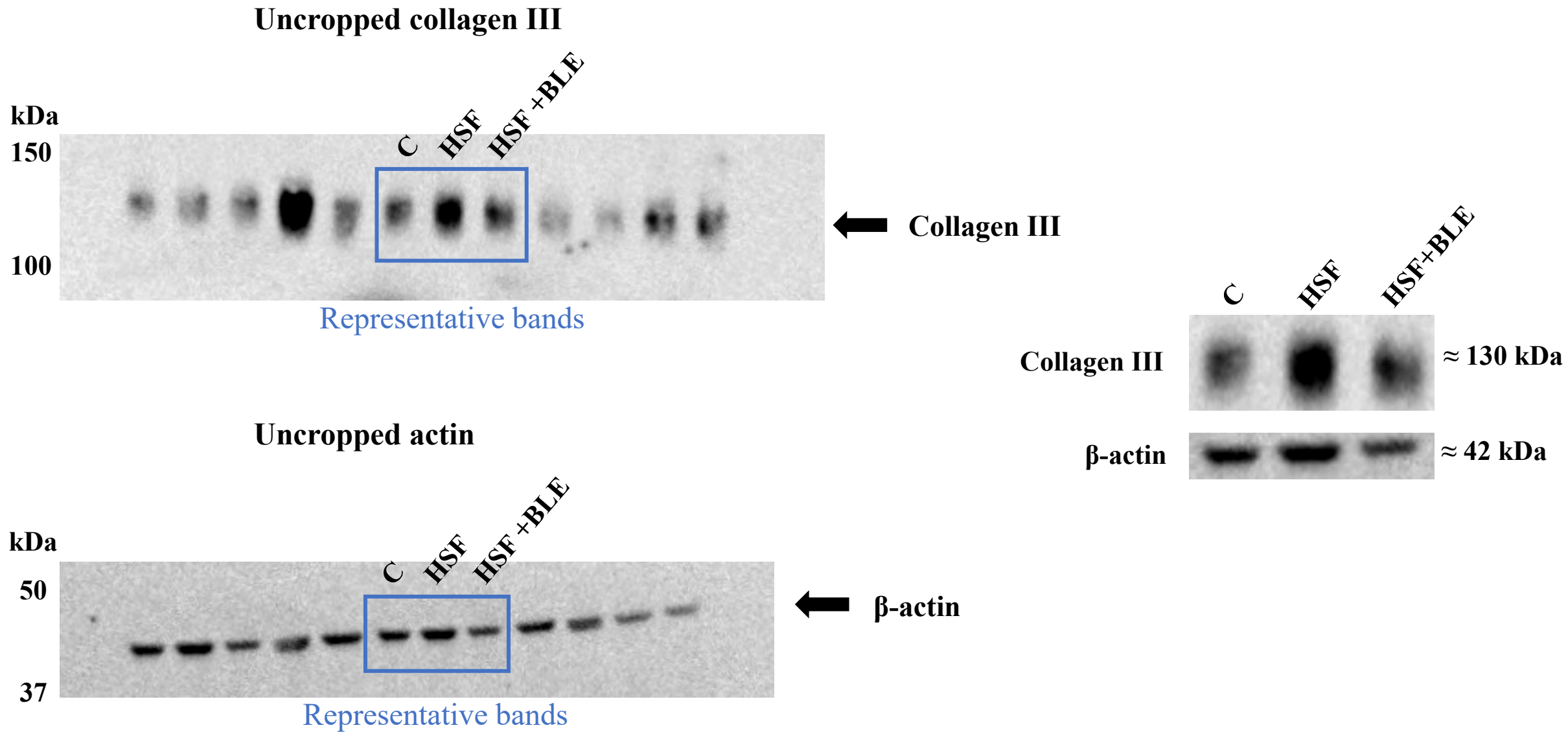

S2 Fig. Raw image 2. Original blots for Fig 3

Supplement: S2 Fig — Raw image 2. Original blots for Fig 3. (PDF) [file pone.0334015.s002.pdf]
